# Zebrafish Models of Paediatric Brain Tumours

**DOI:** 10.3390/ijms23179920

**Published:** 2022-08-31

**Authors:** Faiza Basheer, Poshmaal Dhar, Rasika M. Samarasinghe

**Affiliations:** 1 School of Medicine, Deakin University, Geelong, VIC 3220, Australia; 2 Instiute for Mental and Physical Health and Clinical Translation (IMPACT), Deakin University, Geelong, VIC 3220, Australia

**Keywords:** paediatric brain cancer, zebrafish, animal models

## Abstract

Paediatric brain cancer is the second most common childhood cancer and is the leading cause of cancer-related deaths in children. Despite significant advancements in the treatment modalities and improvements in the 5-year survival rate, it leaves long-term therapy-associated side effects in paediatric patients. Addressing these impairments demands further understanding of the molecularity and heterogeneity of these brain tumours, which can be demonstrated using different animal models of paediatric brain cancer. Here we review the use of zebrafish as potential in vivo models for paediatric brain tumour modelling, as well as catalogue the currently available zebrafish models used to study paediatric brain cancer pathophysiology, and discuss key findings, the unique attributes that these models add, current challenges and therapeutic significance.

## 1. Introduction

Brain and CNS tumours account for 25% of all paediatric cancers and is the second most common form of cancer in children [1,2]. These are some of the most devastating tumours in children due to their highly invasive nature and development within an organ with limited regenerative capacity. Although the 5-year survival rate of patients has improved as a result of multi-modality treatments (including surgery, radiation therapy and chemotherapy), they still leave long-term ongoing and debilitating sequelae, such as infertility, cardiac damage, neurocognitive dysfunction, endocrine defects, visual deficits and poor growth in children [3,4,5,6,7]. Further, children who develop high-grade cancers have poor outcomes and dismal survival [8,9].

**Figure 1:**
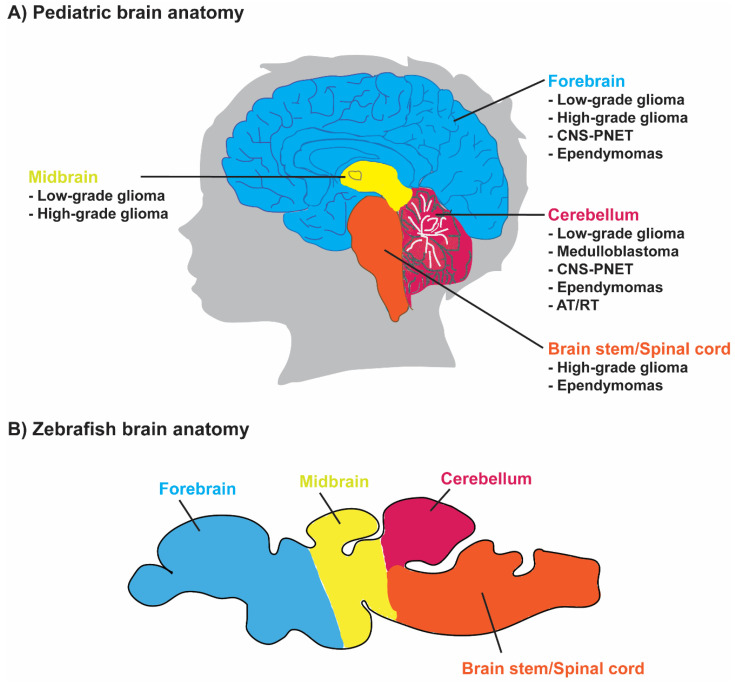
Paediatric brain and CNS tumour locations. (**A**) The paediatric brain tumour types and their location within the brain and CNS and (**B**) zebrafish brain anatomy [28] are shown. CNS-PNET, central nervous system–primitive neuroectodermal tumour; AT/RT, atypical teratoid rhabdoid tumour.

Paediatric brain tumours develop in children of ages ranging from 0–14 years, and have been accounted as one of the leading causes of cancer-related mortality in infants and adolescents [10,11]. The five malignant brain and CNS tumours include high-grade glioma (HGG), ependymomas (EPN), medulloblastomas (MB), atypical teratoid rhabdoid tumours (AT/RT) and primitive neuroectodermal tumours (PNET) [9], which arise at different locations within the paediatric brain (Figure 1). Gliomas are the most common paediatric CNS tumours and represent approximately 47% of all brain tumour cases in children. Gliomas are derived from glial cells, which provide neuronal cell support and within gliomas, 75% of these tumours account for astrocytoma [1,12,13]. These are heterogeneous tumours grading from low-grade gliomas (LGG) to high-grade gliomas (HGG) based on their malignant nature, with their 5-year survival rate varying between 30–90% [14,15]. High-grade gliomas are aggressive infiltrating malignant tumours and are relatively uncommon in children. Patients with HGGs, such as anaplastic astrocytoma (grade III) and glioblastoma (grade IV), have poor prognoses [16]. Another type of paediatric glioma, namely ependymomas, on the other hand, accounts for 6% of all paediatric brain tumours and begins in the radial glial cells of the ependymal lining of ventricles and the central canal [17,18].

Medulloblastoma (MB) is the second largest group of brain tumours (18.8%) and is the most common paediatric embryonal malignant tumour originating from precursor cells in the cerebellum or dorsal brainstem [19]. MBs are heterogenous tumours of a highly proliferative nature and predispose to metastasis. Atypical teratoid rhabdoid tumours (AT/RTs) are very rare, yet highly malignant embryonal CNS tumours and account for 1–2% of all paediatric CNS tumours affecting children aged less than 3 years [20,21]. They originate in the cerebellum as well as in the spinal cord. Primitive neuroectodermal tumours of the central nervous system (CNS-PNETs) are heterogenous brain tumours predominantly observed in young children and adolescents. These are highly aggressive embryonal tumours with poorly differentiated neuroepithelial cells, predominantly located in the cerebrum and rarely occurring in the brain stem and spinal cord [22]. CNS-PNETs account for 3% of paediatric brain tumours with a 5-year survival rate of 50% [23,24,25]. The histological resemblance of CNS-PNETs to medulloblastoma (MB) often results in misdiagnosis and contributes to dismal prognosis [24]. The rarity of CNS-PNETs and the lack of animal models impede the molecular characterization of these highly malignant tumours [26]. CNS-PNETs and MB have a distinct cellular origin and molecular tumour signature, highlighting the need for targeted therapy and animal-based models to test potential drugs. A more detailed review of the molecular and pathophysiology of the paediatric brain tumours is reported in a recent review by Cacciotti et al. 2020 [27].

Children with low-grade brain and CNS tumours have an overall survival rate of 80–90% [29], whereas the 5-year survival rate of high-grade tumours ranges from 15% for gliomas and rhabdoid tumours to 70% for medulloblastoma [9]. Further, the long-term morbidity and surgical resection of tumours cause significant impairment in normal brain development and function [16,25,30,31]. Current treatments include a multimodality approach involving surgery, radiation and chemotherapy. However, the treatment can be highly challenging due to its anatomical location, rendering them inoperable and the development of therapeutic resistance results in treatment failure. Although there are recent advances in the understanding of genomic characterization of paediatric brain tumours, current therapies have been reported to cause long-term health deficits in children, including neurocognitive and neuroendocrine dysfunction [32]. Hence it is critical to characterize and better understand the molecular basis of these paediatric brain tumours using animal-based models that recapitulate the genetic heterogeneity, which will ultimately facilitate in the development of less-invasive and targeted therapies.

Adverse effects imposed by these treatment modalities have raised the need for an alternative targeted approach for paediatric brain cancer treatment, which enhances efficiency with insignificant toxic effects. Advances in next-generation sequencing have led to the advent of an era of precision medicine that catalogues the genetic alterations in patient tumours, helping to understand the molecular mechanisms driving the tumour cell transformation [33,34]. Further, immunotherapy has recently also shown great success with cure rates for paediatric tumours, reduced toxicity and decrease in therapeutic resistance [35,36]. Nevertheless, preclinical evaluation of these therapies is a prerequisite to unravelling the genetic alterations and molecular mechanisms driving the tumorigenesis, and understanding how these could be efficiently targeted, which requires robust animal models that closely mimic the paediatric tumour environment. Such animal models will add invaluable information in designing precisely targeted therapies with improved outcomes for paediatric cancer patients. Mouse models are the cornerstone for in vivo cancer modelling and have helped researchers in understanding the molecular mechanisms of cancer pathogenesis to a certain extent. However, these models hold multiple limitations, such as being costly, performing genetic manipulations on them are labour-intensive and time-consuming, and the in vivo real-time monitoring of tumour formation is difficult and requires sophisticated imaging modalities. Here we describe the critical review of various pre-clinical zebrafish models of paediatric brain tumours, discuss the outstanding challenges associated with them and how these models help in accelerating the identification of novel therapeutics.

## 2. Zebrafish Models of Paediatric Brain Cancer

The zebrafish is a valuable vertebrate model organism initially established for studying developmental biology [37]. Recent advancements in reverse genetics, in vivo imaging techniques, ease of transplantation, high efficiency in transgenesis and genome-editing capabilities have allowed zebrafish to emerge as a robust model for studying cancer biology, including paediatric tumours [38,39]. Zebrafish share a high degree of genetic and physiological homology with humans, with over 70% of all human cancer genes having their functional orthologs in this organism [40]. The molecular mechanisms and key signalling pathways regulating fundamental developmental processes, such as proliferation, differentiation and apoptosis, as well as the candidate disease genes and human cancer-related pathways are conserved between zebrafish and humans [38,41,42,43]. Major attractions of this model include high fecundity, low maintenance costs, optical transparency facilitating the in vivo tracking of tumour growth and progression and the large-scale high-throughput screening of drugs [44,45,46]. Over the last decade, zebrafish have proved their impeccable role in cancer research, where they developed a range of tumours that strikingly resembled both histologically and genetically to human malignancies [47,48,49,50,51].

**Figure 2:**
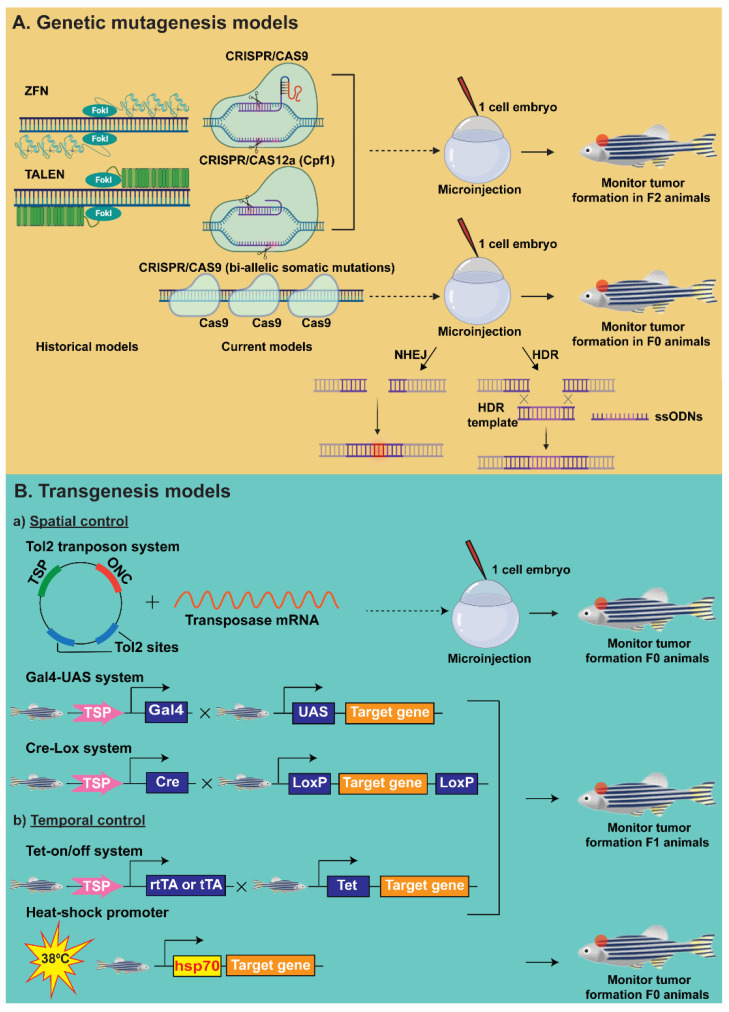

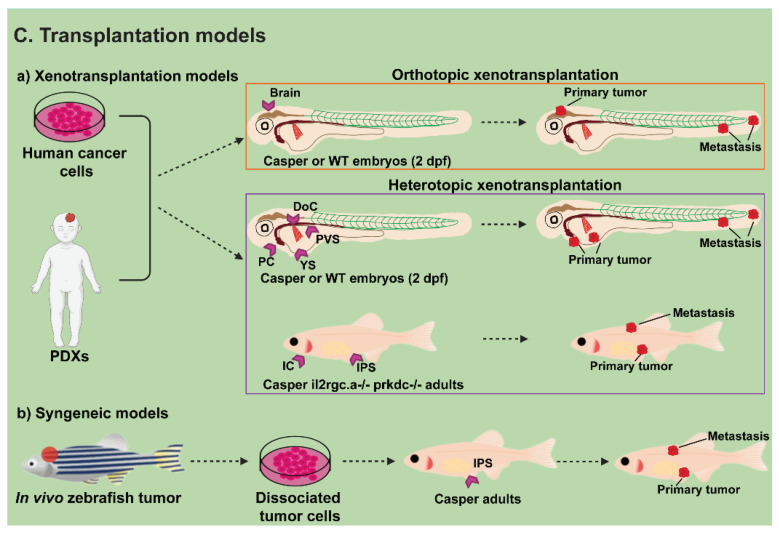
Paediatric cancer modelling in zebrafish involves three main approaches, (**A**) Genetic mutagenesis, (**B**) transgenesis and (**C**) transplantation models. Genetic modelling (**A**) involves the use of multiple techniques, such as historical engineered nucleases, including Zinc Finger Nucleases (ZFNs), Transcription Activator-Like Effector Nucleases (TALENs) and the current generation of engineered nucleases, and Clustered Regularly Interspaced Short Palindromic Repeats (CRISPR)/Cas9 technology with different variants, such as Cas9 and Cas12a, as well as approaches such as multi-targeting to achieve bi-allelic somatic mutations in F0 generation. Transgenesis approach (**B**) allows tumour modelling with spatial (**a**) and temporal (**b**) control on the expression of a target gene or an oncogene (ONC) of interest. Spatial transgenesis techniques, such as tol2 transposon system, Gal4/UAS and Cre-Lox, offer the tissue-specific expression of target gene with the help of a tissue-specific promotor (TSP), where temporal control is offered by Tet-on and Tet-off systems as well as the heat shock promoter, hsp70. Transplantation approach (**C**) involves the injection of human cancer cells or patient-derived xenografts (PDXs) (**a**) into 2 days post-fertilized (dpf) wild-type (WT) or casper embryos and/or immune-deficient casper adults (prkdc^−/−^ il2rgc.a^−/−^) or injecting tumour cells derived from zebrafish brain tumours (**b**) into casper adults to study tumour formation and metastasis. HDR—homology-directed repair, NHEJ—non-homologous end joining, ssODNs—single-stranded oligonucleotides, dpf—days post-fertilization.

Classic methods of cancer modelling in zebrafish involve genetic and transplantation approaches (Figure 2). Genetic approaches include reverse genetic techniques, such as genome editing or mutagenesis, as well as transgenesis, facilitating the creation of gene-targeted mutations that can create loss-of-function of an important tumour suppressive gene or the generation of a stable transgene that overexpresses an oncogene of interest, whereas transplantation involves the implantation of human cancer cells into an in vivo model organism. Zebrafish serve as an ideal alternative to mouse models as they offer greater efficiency and ease for genetic modifications to be performed, as well as facilitate combinatorial functional studies of multiple genes by creating or combining multiple genetic variants [52,53,54]. Zebrafish transgenic lines that express a fluorescent protein in a tissue-specific manner have been exploited to provide further insights into tumour biology—tumour growth, dissemination, the dynamics of tumour pathogenesis and tumour micro-environment at the molecular level in real-time [55,56,57].

Zebrafish embryo xenotransplantation models have been widely used to understand the key steps of tumour progression, such as invasion [58,59], extravasation [60,61], angiogenesis [62,63,64,65], cancer stem cell renewal [45,66] and the formation of micrometastatic lesions [56,65,67,68]. They have a delayed adaptive immune system, with T and NK cells developing at around 5 dpf and B cells by 21 dpf, which become completely functional by 3 weeks post-fertilisation, allowing a short window for human cancer xenograft studies to be performed without any immune rejection [69,70]. The optical clarity of zebrafish larvae, along with the advent of genetically modified adult zebrafish lacking pigmentation, “Casper” strains add greater attraction, as this allows the real-time in vivo monitoring of early stages of cancer development and disease pathogenesis [71]. A foremost limiting factor for transplantation studies in zebrafish includes the ambient temperature variation between human and zebrafish cells, where human cells grow optimum at 37 °C [72], whereas zebrafish are maintained at 28 °C [73]. However, a recent study has developed a novel immune-deficient zebrafish model casper prkdc^−/−^ il2rgc.a^−/−^, which lacks B, T and NK cells, allowing xenotransplantation studies to be performed in adult animals and has reported ways to induce tolerance to higher temperatures in these animals [74]. Collectively, progress in genome editing, transgenesis, protein knockdown and overexpression, cancer xenotransplantation, chemical screening and imaging techniques has dramatically altered the landscape of this model organism to a reliable and unique model for unravelling the mechanisms of paediatric cancer development and progression.

### 2.1. Genetic Mutagenesis Models

The advent of engineered nucleases has portended a booming growth in the field of reverse genetics in modelling cancer in zebrafish [75] (Table 1). The engineered nucleases have been widely used to create loss-of-function and gain-of-function alleles as well as to create conditional gene regulation. Zinc Finger Nucleases (ZFNs) were the first method that was developed for targeted genome editing in zebrafish [76]. ZFNs are composed of a site-specific DNA-binding domain and a FokI endonuclease domain, which facilitate the introduction of a DNA double-stranded break (DSB). A major limitation imposed by this technique is off-targeting, which resulted in surpassing this technique with Transcription Activator-Like Effector Nucleases (TALENs) [77]. TALENs are protein-based editing tools and similar to ZFNs, which possess a DNA-binding domain and a FokI endonuclease domain. TALENs are more reliable and efficient than ZFNs, offer better target specificity and have been successful in generating a wide variety of mutations in zebrafish [78,79,80,81,82]. As with ZFNs, TALENs are expensive and time-consuming with significant off-target effects curtailing their widespread use, which led to the current generation of targeted genome-editing guided by Clustered Regularly Interspaced Short Palindromic Repeats (CRISPR)/Cas9 technology [83] (Figure 2A). CRISPR/Cas9 system is composed of a short single-guide RNA (sgRNA), which is complementary to the 20-base pair (bp) target genomic sequence and Cas9 endonuclease. The 20 bp target sequence is followed by a protospacer adjacent motif (PAM) sequence, NGG, which is essential for the Cas9 endonuclease to work. The sgRNA mediates the binding of Cas9 to the target site, resulting in the generation of a DNA DSB, which is either repaired by non-homologous end joining (NHEJ) or homology directed repair (HDR) with the aid of a repair template facilitating targeted DNA integration. Among the HDR template, asymmetric anti-sense single-stranded oligonucleotides (ssODNs) have been reported to be highly efficient in generating specific knockin point mutations when used in combination with CRISPR/Cas9 system, enabling the precise recapitulation of disease-causing mutations [84]. These editing tools allow us to recreate the oncogenic events in patients by facilitating the generation of oncogenic mutations, and with the help of HDR, allow in mimicking fusion protein expression and/or chromosomal rearrangements [85,86,87]. Of these genome editing tools, CRISPR/Cas9 system has been proven to be pre-eminent in generating specific mutations and for targeting multiple genes with great precision, ease and high efficiency [88,89]. CRISPR/Cas9 system has evolved expeditiously over the last few years with the development of numerous zebrafish cancer studies employing this system in the rapid testing of cancer-associated potential identifiers or driver genes in a tissue-specific manner. Thus far, many of these engineered genetic models that express an oncogene or incorporate inactivation in tumour suppressors have been successful in modelling human malignancies sharing similar disease-specific molecular characteristics and analogous cancer signalling pathways [90,91,92,93]. The current progress in this technique has expanded its wide application to epigenome editing, lineage tracing, transcriptional modulation and the live imaging of the genome [88,94,95,96]. CRISPR/Cas9 system requires the NGG PAM motif near the target site for nuclease activity, which limits the targeting scope of this editing tool. Efforts to improve the target coverage and specificity of Cas9 enzymes have resulted in the generation of modified CRISPR systems. These include the Cas12a (Cpf1), xCas9 and SpCas9-NG, which have been engineered to have different PAM specificities, for example, Cpf1 recognizes T-rich PAM [97], xCas9 a broad range of PAMs (NG, NGG, CAA, GAT and GAA) [98] and spCas9 allows the relaxed preference for their third nucleobases in the NGG (NGA, NGT and NGG) [99]. These modified Cas nucleases have been successful in expanding the target coverage and improving the specificity [88,98,100,101]. A recent study also reports the use of a simple CRISPR/Cas9 system for the efficient generation of biallelic mutations in the zebrafish F0 generation. This approach involves the use of three synthetic gRNAs targeting multiple loci within the gene for greater precision and efficiency. This approach is highly beneficial as it allows genetic screens to be performed rapidly making them a robust system for generating F0 knockouts and for studying the disease or other developmental phenotypes within a week [102]. In addition to these engineered nucleases, other historical techniques, such as morpholino oligonucleotides (MO) and RNAi, have been used previously for genetic modifications but to a lesser extent due to their incomplete knockdown and non-specific off-target effects [103,104,105]. A comprehensive resource discussing the genetic approaches employed in zebrafish cancer research has been published by Rafferty SA et al. and Ruby L et al. [106,107].

Studies using MOs have been used to unravel the molecular mechanisms of glioblastoma pathogenesis and to identify potential therapeutics. The MO-mediated knockdown of Ephrin-B3 ortholog decreased tumour vascularization, facilitated EphA4-induced cell death and thereby decelerated the GBM growth in zebrafish [108]. Similarly, the knockdown of Plexin-A1 in the zebrafish GBM model revealed its role in inducing both developmental and GBM tumour-specific pro-angiogenesis, identifying Plexin-A1 as a potential therapeutic target for treating GBM by blocking the pro-angiogenic effects [109]. ZFNs have been used to generate the first zebrafish genetic model of paediatric brain cancer. It created the loss-of-function alleles of *nf1a* and *nf1b*, *NF1* orthologues in zebrafish, which accelerated the tumour onset and increased the penetrance of high-grade gliomas and malignant peripheral nerve sheath tumours (MPNSTs), indicating the tumour suppressive role of nf1 in zebrafish [110]. Further, the CRISPR-Cas9 mediated loss-of-function mutations in *atrx* in the abovementioned nf1/p53-deficient zebrafish background resulted in the onset of a broad spectrum of sarcomas and other carcinomas [111].

The TALEN-mediated somatic inactivation of zebrafish retinoblastoma 1 (rb1) tumour suppressor predominantly induces CNS-PNET tumours at a high frequency. The histological analysis of these tumours revealed a lack of neuronal differentiation markers (HuC/D and SV2) indicating the highly infiltrating feature of the tumour [112]. The use of TALENs in this study to generate the targeted somatic inactivation of the rb1 tumour suppressor in G0 mosaic animals helped in understanding their role in driving tumorigenesis, without causing embryonic lethality. The same group later used this *rb1* mutant CNS-PNET model to understand the epigenetic regulators driving oncogenesis in this zebrafish brain tumour model. An extended RNA sequencing analysis of *rb1* somatic inactivation-induced brain tumours revealed the strong expression of oligo-neural differentiation markers *olig2*, *sox10* and *sox8b*, consistent with those observed with human CNS-PNETs. The comparative transcriptome analysis of somatic *rb1* tumours with germline *rb1/rb1* homozygous mutant tissues revealed the overexpression of chromatin remodellers *histone deacetylase 1* (*hdac1*) and *retinoblastoma binding protein 4* (*rbbp4*) in *rb1* tumours. These epigenetic regulators are identified to be driving the embryonal brain tumour pathogenesis by facilitating neural stem cell/progenitor proliferation and survival [113]. Although CNS-PNETs histologically resemble medulloblastoma, RNA sequencing analysis revealed the distinct cellular origin and molecular signature of these tumour entities. Medulloblastoma has a significantly higher expression of proneural gene NEUROG1 and neurogenic transcription factors ATOH1, ATOH2 and ATOH3, while CNS-PNETs express higher levels of neural stem marker SOX10. This study revealed the lack of expression of *neurog1*, *atoh1a*, *atoh1b* and *atoh1c* and the elevated expression of *sox2* in *rb1*-deficient brain tumours [114]. Along the same lines, Shim J et al. 2017 reported the generation of a PNET model by the TALEN-mediated somatic inactivation of *rb1* or *cdkn2a/b* tumour suppressor genes in zebrafish. The somatic inactivation of *rb1* induced MB, such as PNETs, with tumour incidence accelerated from 23.3% to 57.5% when the *rb1* gene was inactivated in the *p53* mutant background. The molecular characterization of these tumours showed the upregulation of genes to be important for neuronal development, cell-cycle progression and protein synthesis. However, the somatic inactivation of *cdkn2a/b*-induced MPNSTs in zebrafish [115]. In another study aimed at characterizing the ENU mutagenesis-based zebrafish knockout mutants of three MMR genes, *mlh1*, *msh2* and *msh6*, in addition to neurofibromas, some of the animals developed PNETs by 6 months of age [116]. 

**Table 1:** Genetic models of paediatric brain tumours in zebrafish.

Approach	Cancer	Genetic/Transgenic Approach	Gene/Protein	Zebrafish Strain	Generation	Ref.
Knockout	CNS PNETs	CRISPR/Cas9/TALEN	*rb1*, *rbbp4* and *hdac1*	WT/Tg(*H2A.F/Z*-GFP)	F0 mosaic adults, heterozygote and homozygote embryos	[113]
		ENU	*mlh1/msh2/msh6*	WT	Heterozygote adults	[116]
		TALEN	rb1	WT	F0 mosaic adults	[112]
		TALEN	*rb1/cdkn2a/b*	tp53^M214K^	F0 mosaic adults	[115]
Knockout	Glioblastoma	CRISPR/Cas9	*atrx*	WT/Tg(gata1:GFP)/*p53^−/−^/nf1^−/−^*	Heterozygote and homozygote embryos and adults	[111]
		Morpholino	Ephrin-B3/EphA4	Tg(*fli*:EGFP)	F0 embryos	[108]
		Morpholino	Plexin-A1	Tg(*kdrl*:eGFP)	F0 embryos	[109]
		ZFN	*nf1a/nf1b*	Tg(*gfap*:GFP)/Tg(*sox10*:GFP)/Tg(*olig2*:GFP)/*p53*^−/−^	Heterozygote and homozygote double knockout embryos and adults	[110]
Transgenesis	CNS PNETs	I-SceI meganuclease-mediated	NRAS	Tg(*sox10*:mCherry-NRASWT)/p53^M214K^ Tg(*sox10*:mCherry-NRASQ61R)/p53^M214K^	F0 mosaic adults	[50]
		Tol2 system (ubiquitous expression)	PAX3-FOXO1	Tg(*BetaActin*-GFP2A-PAX3FOXO1)	F0 mosaic embryos adults	[117]
Transgenesis	Glioblastoma	Gal4-UAS	*ptf1a*/Rac1/Akt1	Tg(UAS:*myrAKT1*; *ptf1a*:Gal4-VP16)/ Tg(UAS:GFP-RAC1^G12V^; *ptf1a*:Gal4-VP16)	Stable transgenic embryos and adults	[118]
		Gal4-UAS	HRAS/YAP	Tg(UAS:GFP-HRAS^G12V^; *zic4*:Gal4-VP16)/Tg(UAS:YAP^S5A^)	F0 mosaic and stable transgenic embryos and adults	[119]
		Gal4VP16-UAS binary transgenic	Smoa1/AKT1	Tg(UAS:*smoa1*-GFP; *krt4*:Gal4-V16)/Tg(UAS:*myrhAKT1*)	Stable transgenic embryos and adults	[120]
		Gal4VP16-UAS binary transgenic	Smoa1	Tg(UAS:*smoa1*-GFP; *krt5*:Gal4-VP16)	F0 mosaic and stable transgenic adults	[121]
		TetOn (Doxycycline inducible)/Gal4VP16-UAS	KRAS	Tg(UAS:mCherry-KRAS^G12V^; *krt5/gfap*:Gal4-VP16)/ Tg(TRE:mCherry-KRAS^G12V^; *krt5/gfap*:rtTa)	Stable transgenic embryos and adults	[122]
		Tol2 (tissue-specific promoter)	IDH1	Tg(*nestin*: eGFP-IDH1^wildtype^; IDH1^R132H^; IDH1^G70D^; IDH1^R132C^) Tg(*gfap*: eGFP-IDH1^wildtype^; IDH1^R132H^; IDH1^G70D^; IDH1^R132C^) Tg(*gata2*: eGFP-IDH1^wildtype^; IDH1^R132H^; IDH1^G70D^; IDH1^R132C^)	Stable transgenic embryos	[123]
		Tol2 (tissue-specific promoter)/LexPR transcriptional activator	AKT1/*cxcr4*	pDEST-lexOP:AKT1/pDEST-lexOP:AKT1/ *cxcr4b^−/−^* mutant	F0 mosaic embryos	[124]
Transgenesis	Medulloblastoma	Gal4-UAS	KRAS	Tg(*ptf1a*:Gal4)/UAS:eGFP-KRAS^G12D^	F0 mosaic embryos adults	[125]

### 2.2. Transgenic Models

The transparency of zebrafish embryos and the optically clear adult casper lines have escalated the diverse potential of this model in generating transgenic zebrafish lines that express fluorescent tissue-specific proteins or express mammalian oncogenes under a zebrafish tissue-specific promoter [49,71,83,126,127]. These models not only facilitate the in vivo monitoring of basic developmental processes, such as cell division, migration and differentiation, but allows the in vivo real-time tracking of cancer development and pathogenesis, aiding in the search for novel therapeutics [93,128,129,130,131,132]. Zebrafish are well-suited for genetic manipulation and the relative ease with which a foreign DNA can be introduced and expressed in zebrafish cells [106]. Transgenic zebrafish lines can be generated to specifically mark a certain lineage to understand the key processes associated with the respective cell lineage. For example, the transgenic line Tg(*fli1*:GFP) is one of the first stable zebrafish transgenic lines generated, which labels their entire vasculature system with green fluorescence [133]. These fish are of high value in the current era of cancer biology as this allows for in vivo tracking the key step ‘angiogenesis’, the formation of new blood vasculature, in the metastatic growth and spread of the tumour [65,67,134,135,136,137,138]. Other transgenic models, such as Tg(*mpx*:GFP), which marks neutrophils, and Tg(*mpeg*:GFP), which marks macrophages, have been used for innate immunity studies as well as to understand these immune cell interactions with the tumour microenvironment [56,139,140]. In addition, zebrafish cell and tissue-specific reporter lines have also been widely used for understanding different tumour biology and characteristics, such as tumour cell growth, invasion, metastasis, angiogenesis and drug response [125,141,142,143,144]. The transgenesis field has significantly advanced in recent years and currently this allows conditional transgenic approaches with spatial and temporal control in the expression of the desired gene (Figure 2B). Spatial control offers a cell-type specific expression of the gene rather than ubiquitous gene expression, whereas temporal control offers control over the window during which a gene is expressed or functions. The transgenesis tools that offer spatial and temporal control over an oncogene expression is crucial for cancer modelling in zebrafish. Transgenic techniques that offer spatial control include SceI-mediated transgenesis, *Gal4/UAS* system and site-specific recombinases, such as Cre-loxP, Flp/Frt, Dre/rox and phiC31 systems [145,146,147,148,149]. SceI is a meganuclease that recognizes a unique 18-bp sequence, which is absent in the zebrafish genome and promotes transgenesis by cleaving the two I-SceI meganuclease recognition sites flanking the transgene of interest [150]. An alternative to this is the Tol2-based transposon system, consisting of a transposon donor plasmid carrying the transgenic cassette flanked by cis-regulatory repeats, which are important for driving transposition and an in vitro transcribed transposase mRNA [151]. Once injected into the zebrafish embryos, Tol2 is excised from the donor plasmid and integrated into the genome of the germ lineage facilitating germline transmission of the transgene expression [152,153]. Gal4/UAS system is one of the first conditional transgenesis techniques used in zebrafish [154]. Gal4 is a transcriptional activator that controls gene expression by binding to its Upstream Activating Sequence (UAS) element, which is the DNA-binding motif [155]. This system facilitates tissue-specific expression by placing Gal4 under the control of a tissue-specific promoter and the transgene of interest downstream of UAS. A key advantage of this system is the feasibility of maintaining the two components as separate lines, the driver line expressing Gal4 and the effector line expressing UAS, thereby facilitating the silent inheritance and conditional expression of lethal or toxic genes only in the double-transgenic offspring. Capitalizing on Gal4-UAS- and Tol2-transposon-based systems, many zebrafish models of paediatric cancers have been generated. Cre-loxP is the most commonly used site-specific recombinase system [156,157]. Cre recognizes and induces recombination at specific 34 bp target sites called loxP sites, which are usually flanked on both ends of the gene of interest. Depending on the orientation of the two loxP sites, Cre recombination induces different DNA rearrangement events. Cre/loxP system has enabled researchers to efficiently induce conditional gene/oncogene expression, create genetic knockouts, lineage tracing and for inducing chromosomal rearrangements [148,158,159,160,161,162,163]. Similar to the Gal4/UAS system, Cre/loxP system also allows the maintenance of separate zebrafish lines that express Cre recombinase under a tissue-specific promoter and a loxP flanked gene of interest line, facilitating the tight regulation of the transgene expression [156,157]. Along the same line, Flp/frt acts as an alternative site-specific recombinase, where Flipase (Flp) recognizes and cleaves two frt sequences in an analogous fashion to the Cre/loxP system. Cre and Flp exclusively recognize and cut loxP and frt recognition sites respectively, and therefore both systems could be used in combination in a genetic line to enhance the transgenic capabilities [156,164]. Dre/rox is the third recombination system, wherein Dre recognizes sequences called rox that resemble the loxP sites but are not compatible with Cre [165]. Whereas the PhiC31 system is derived from PhiC31 bacteriophage and offers better spatial control compared to other transgene integration. The recombination is highly specific and occurs between the attP and attB sites, resulting in hybrid attL and attR sites, without any additional requirement of co-factors [166]. Temporal control in transgenesis is facilitated by the use of heat shock promoters, tamoxifen, Tet-On, Tet-Off systems and optogenetics [58,122,167,168,169,170,171,172,173,174,175]. Heat shock protein 70 (hsp70) is the widely used heat shock promoter in zebrafish, which mediates temperature-regulated expression of a transgene in a time-controlled fashion. Zebrafish hsp70 consists of tandem repeats of 5 bp DNA consensus sequences called heat shock elements (HSE), which are activated by the transcription factor heat shock factor (HSF), in response to an increase in temperature [176,177]. However, this technique imposes a major drawback of the leakiness of hsp70, where non-heat-shocked double transgenics lines displayed transgene expression. In addition, heat shock induction in embryos is limited due to the adverse effects on zebrafish embryonic development [178]. The tetracycline (Tet) system controls the expression of genes involved in tetracycline resistance. Tetracycline repressor (tetR) reacts with the tetracycline or to its more stable derivative doxycycline (Dox) in mediating its effects. A Dox-controlled transactivator (tTA) is generated by the fusion of tetR to the transactivation domain of transcription factor VP16. Here in the TET-Off system, the absence of Dox mediates the binding of tTA to the tetracycline operator (tetO) and activates transcription from a minimal promoter, whereas Dox binding inhibits tTA transcriptional activation [179]. Tet-On system on the other hand relies on a mutant transactivator (rtTA) that is inactive in the absence of Dox and can bind to tetO promoters activating transcription only upon Dox binding [180]. Finally, optogenetics relies on light-gated proteins for the targeted control of cellular behaviour and has been shown to be effective in mediating the precise spatial and temporal control of the stimulation and inhibition of cellular activities [181]. Optogenetics has limited application in cancer research. A comprehensive overview of conditional transgenesis approaches employed in zebrafish cancer modelling has been reviewed in an article published by Mayrhofer and Mione [156]. Although many of these transgenesis techniques offer great advantages for the spatial and temporal control of transgene expression, several of these techniques are less explored in zebrafish paediatric brain tumour modelling. So here in this review, we focus on the key transgenesis techniques currently employed for paediatric cancer modelling in zebrafish (Figure 2B).

Recent years have witnessed the generation of a variety of zebrafish paediatric tumour models that have been exploited for unravelling the molecular mechanisms driving tumour formation. A transgenic model of zebrafish glioblastoma was generated by Ju et al. in 2009, by employing a binary transgenic approach *Gal4VP16-UAS* system, which co-expressed an oncogenic zebrafish Smoa1 with a constitutively active human AKT1, which induced serval types of tumours with a higher incidence of glioblastoma and astrocytoma [120]. The same group later developed a zebrafish model of Shh signalling-driven gliomagenesis by inducing an ectopic expression of Smoa1 in neural progenitor cells driven by zebrafish *cytokeratin 5* (*krt5*) gene promoter using a similar *Gal4VP16-UAS* approach. These animals developed various retinal tumours and optic pathway gliomas [121]. Further, they induced the transient transgenic expression of human oncogenic KRAS, KRAS^G12V^ in putative neural stem and progenitor cells driven by *krt5* and *gfap* promoters by employing a doxycycline-inducible Tet-On/Gal4VP16-UAS system, which resulted in the formation of canonical Ras and mTOR pathway-driven malignant brain tumours in the cranial cavity and parenchyma, respectively [122]. Jung IH in 2013 reported a Gal4/UAS system-based gliomagenesis model, generated by overexpressing dominant-active (DA) human Akt1 (DAAkt1) or Rac1^G12V^ (DARac1) under the ptf1 promoter. The induction of DAAkt1 alone induced glioma in the cerebellum at a higher frequency of about 36.6–49% over the period of 6 to 9 months. Although DARac1 alone did not induce gliomagenesis, co-expression of DARac1 with DAAkt1 induced gliomagenesis at an accelerated rate with higher incidence (62% at 6 months and 73.3% at 9 months), progressed histological grade and highly invasive tumours. Results indicated that DARac1 accelerates gliomagenesis by enhancing epithelial-to-mesenchymal transition (EMT), proliferation and survival [118]. In another study, a zebrafish model of brain tumours was developed using a Gal4/UAS system to induce the somatic expression of oncogenes, such as HRAS^G12V^ or YAP^S5A^, KRAS^G12V^, AKT, EGFR^vIII^ and BRAF^V600E^ under the control of *zic4* enhancer, which activates MAPK and PI3K signalling pathways. Oncogenic RAS induced aggressive brain tumours and/or heterotopias in zebrafish, with persistence in signal determining the occurrence of benign or aggressive tumours. These aggressive tumours resembled the human mesenchymal GBM with a strong YAP component, validating YAP activation as a critical hallmark for determining malignant brain cancer. This model provides a strong platform for performing pre-clinical studies and drug screening that could prevent malignant transformation of the GBM subtype [119]. This GBM zebrafish model was later used by Idilli et al. in 2020 to understand the telomere maintenance mechanism in paediatric brain cancer progression [182]. Further, zebrafish transgenic models that express a range of frequent glioma missense mutations in IDH1 gene were created by the Tol2 tissue-specific promoter-driven system to assess their role in tumour formation. However, these mutants did not develop any tumours indicating the need for additional transforming events for glioma pathogenesis [123]. Recently, a study reported the generation of a zebrafish transgenic line using a Tol2/LexPR transcription activator system that expresses human AKT1 under a neural-specific beta tubulin (NBT) promoter, which induced brain tumours with increased microglia population in neural cells mediated by Sdf1b-Cxcr4b signalling. This model validated the tumour-promoting functions of macrophages and microglia during the early stages of tumour microenvironment development [124].

Based on comparative genomic analysis, CNS-PNETs are classified into three distinct sub-groups, namely primitive-neuronal, oligoneural and mesenchymal. Oligoneural subtype, CNS NB forkhead box R2 (NB-FOXR2) is characterised by the elevated expression of oligodendrocyte precursor cell (OPC) genes *SOX10* and *OLIG2*. Based on this, a zebrafish model of CNS NB-FOXR2 was generated using an I-SceI-mediated transgenesis system by activating NRAS in Olig2 and Sox10 expressing OPCs in homozygous p53^M214K^; mitfa^w2^ embryos (lacking p53 activity and deficient in pigment (melanophore)), which displayed brain tumour onset by 6 weeks post fertilisation (wpf). Genomic analysis and histological studies on the tumour sections showed similar histopathology and cell morphology to human CNS NB-FOXR2. The drug response of zebrafish CNS NB-FOXR2 tumours to MEK inhibitor (AZD6244) showed promising effects on tumour burden decline and increase in overall survival [50]. Further, in a recent study evaluating the role of PAX3-FOXO1 fusion oncogene in alveolar rhabdomyosarcoma, the beta-actin promoter-driven expression of this fusion oncogene mediated by Tol2 transposase system induced PNETs in zebrafish brain by 3 months of age in 5% of the injected animals. This study reports HES3 transcription factor, which is important for the developing brain and inhibits neural stem cell differentiation as a mediator for oncogenesis [117]. 

The Mebulloblastoma (MB) model of zebrafish was developed based on a transgenic Gal4-UAS system using the promoter and enhancer elements of ptf1a to drive the expression of KRAS^G12V^ in cerebellar GABAergic neurons. The expression of oncogenic KRAS^G12V^ in exocrine pancreas induced pancreatic adenocarcinoma in zebrafish. Zebrafish MB model displayed the dysregulation of Smad3/TGFβ, Shh and Notch pathways during MB pathogenesis, with the inhibition of Notch signalling during the early stages of MB development and the upregulation of TGFβ and Shh pathways during MB development and carcinogenesis. This study elevated the potential of coupling zebrafish cancer models with fluorescent reporter lines in the in vivo tracking of hallmark signalling pathway components contributing to tumorigenesis [125]. 

### 2.3. Transplantation Models

The transplantation of tumour cells into zebrafish has emerged as a robust platform for unravelling the mechanisms driving tumour cell initiation, progression, invasion, angiogenesis, metastasis, interaction with tumour microenvironment and therapy response. Zebrafish xenografts have been proven to engraft and develop tumours that share similar histopathology and molecular features to human cancers [39,183]. The tumour cells can be either derived directly from patients as primary cells or be based on the laboratory cell lines (Figure 2C(a)). Patient-derived xenografts (PDXs) are established by the transplantation of tumour cells derived directly from the resected patient tumour or biopsies. Although PDXs offer a better understanding of the cancer biology as it retains the patient’s intrinsic tumour heterogeneity and biological profile, it poses a major concern as the implantation demands large number of tumour cells as well as the extended time required for the tumour engraftments to grow [184]. Mouse models are the gold standard for performing human cancer cell transplantation studies as they recapitulate tumours retaining the genetic and epigenetic complexity similar to those of patients [184,185,186,187]. However, murine xenografts need a large number of patient samples for engraftment as well as requiring extended time from weeks to months for establishing tumours [188]. In addition, the large-scale use of this animal model is expensive, and it is not suitable for the in vivo tracking of transplanted tumour cells, which is only possible by creating surgical imaging windows for long-term intravital imaging and using multi-photon imaging techniques [189]. Zebrafish xenografts moderate these limitations as they only take days to weeks to establish tumours [188] as well as allowing the in vivo tracking of the engrafted cells at high resolution, helping to unravel the mechanisms driving the hallmarks of cancer [39]. These models offer many advantages over mouse xenografts as they facilitate the real-time monitoring of complex, multi-step processes involved with tumour progression and spread, such as invasion, intravasation, extravasation, metastasis and/or secondary tumour colonization [62,190,191,192,193,194,195].

Zebrafish embryos are extensively used for xenotransplantation studies as they develop adaptive immunity, including the generation of T, B and NK cell repertoires after 7 dpf, which are not fully functional by 3 weeks, providing a short window of up to 7–10 days for studying the tumour invasion and metastasis of human cancer cells, including those that are derived from patients [69,196,197,198]. The anatomical site of cancer cell implantation varies with each cancer cell and the study type. Tumour cells can be transplanted into a zebrafish tissue/organ similar to the primary location of the patient tumour, resulting in orthotopic xenograft models (Figure 2C(a)). These orthotopic xenografts help in recapitulating the tumour heterogeneity and molecular features as seen in patients and aid in unravelling the tumour microenvironment interactions and signalling pathways involved in tumour pathogenesis. On the other hand, heterotopic xenografts are derived through the implantation of cancer cells into other injection sites, such as the yolk sac, perivitelline space or the duct of Curvier in zebrafish embryos, and into the intraperitoneal cavity in adult xenografts [43,199,200,201,202,203,204,205,206,207]. Heterotopic xenograft models are widely used to monitor cancer cell engraftment, survival, proliferation and metastasis and to test the drug efficacy in tumour regression and clearance. Further, transplantation can also be performed in an allogeneic fashion, where the tumour cells are derived from an established zebrafish model, allowing transplantation into an immune-competent zebrafish, resulting in syngeneic models (Figure 2C(b)). These syngeneic models are beneficial as they facilitate long-term tumour cell engraftment and allow studies on interactions between immune cells and the tumour microenvironment [208]. Xenotransplanted cells are usually labelled with a fluorescent cell labelling dye, such as Dil, prior to the implantation and the optical clarity of embryos as well as the pigment-deficient “Casper” strain facilitates real-time visualisation and evaluation by fluorescence or confocal microscopy [39,71,209,210]. These xenografts are maintained at 32–34 °C to mimic human body conditions. Efforts to perform xenotransplantation in adult zebrafish resulted in the generation of immune-deficient animals that lack T, B and natural killer (NK) cells obtained by creating a compound mutant strain, Casper il2rgc.a^−/−^ prkdc^−/−^ zebrafish line [74]. These animals showed the stable engraftment of a range of human cancers with analogous growth kinetics and histology to those observed in mice and facilitated the therapeutic testing of drugs with dosages that are clinically relevant [74]. Transgenic zebrafish xenograft models are useful in studies aimed at understanding the tumour microenvironment interactions as well as help in exploring mechanisms driving neovascularisation. For example, Tg(*fli1*:GFP) zebrafish line with GFP-labelled vasculature, in particular, helps in understanding the mechanisms of neovascularisation, a crucial step in the tumour progression process that supports the growing tumour, as well as allowing therapeutic testing of antiangiogenic agents to inhibit tumour neovascularisation and thereby prevent tumour progression [67,135,136,137,138,211]. Tumour microenvironment plays a central role in tumour initiation, growth, progression and metastasis. Immune cells, such as neutrophils and macrophages, have been reported to initiate the early events of metastasis through their interactions with cancer cells [212,213,214,215,216,217,218,219,220]. Transgenic zebrafish lines, such as Tg(*mpx*:GFP) and Tg(*mpeg*:GFP), have been used to determine the influence of neutrophils and macrophages in driving tumour metastasis [55,57,221]. In addition, the xenotransplantation of cancer cells in different genetic backgrounds has aided in defining the role played by these genes in tumour cell growth and progression [222]. Collectively, zebrafish PDXs and xenograft models have added valuable information to the understanding of human cancer biology, offered a great platform for performing preclinical trials and provides therapeutic choices for personalised medicine.

Investigations in paediatric brain cancer biology and associated novel therapeutics have marked a significant milestone with the help of zebrafish xenograft models. Several studies have modelled human brain tumours, such as glioblastoma and medulloblastoma using zebrafish embryonic xenografts to study tumour growth, angiogenesis, cellular interactions and response to anticancer therapies (Table 2). Although different studies adopted varying sites for brain tumour implantation in zebrafish embryos, orthotopic transplantation of brain tumour cells into the zebrafish brain ventricles or hindbrain–midbrain boundary has recently gained greater attention as it better replicates the human tumour microenvironment, tumour–host interactions and pathogenesis. Pudelko et al., in 2018, reported a rapid and robust automatable transplantation approach in generating orthotopic xenografts, where the cells are injected into the blastula stage of embryonic development, which later migrates into the developing nervous system, resulting in intracranial engraftment of tumour cells by 24 hpi [195]. However, it was in 2015 that Eden C. J. for the first time demonstrated the generation of orthotopic xenograft models of paediatric brain tumours in zebrafish. Mouse glioma, ependymoma and choroid plexus carcinoma cells expressing red fluorescence protein were implanted into the cerebrum via the intranasal route of a 30-day-old, immunosuppressed zebrafish. Zebrafish xenografts recapitulated the biology and histology of the mouse tumour and validated the potential of these orthotopic xenografts in preclinical drug testing [223]. Based on these findings, a paediatric CNS-PNET and a human glioma model of orthotopic xenografts were established for drug screening by the transplantation of tumour cells into the fourth ventricle and midbrain of zebrafish embryos, respectively [50,224]. The paediatric CNS-PNET model displayed high efficiency in tumour engraftment and confirmed MEK inhibitors as an effective therapeutic approach for treating children with embryonal tumours expressing *SOX10* and *OLIG2* [50]. Casey and colleagues, in 2017, reported a syngeneic orthotopic transplantation approach, where the paediatric CNS-PNET tumour cells isolated from an established zebrafish tumour model were transplanted into an immune-competent host to evaluate the tumour cell behaviour and drug response over an extended period of time. These syngeneic models are ideal for studying tumour cell interactions, host–immune responses, tumour heterogeneity, metastatic properties and drug responses [225]. Further, orthotopic the paediatric brain tumour xenografts of LGG have been established for determining the tumour cell survival and migration within zebrafish brain [226]. On the other hand, others used stem cell cultures derived from high-grade glioma or glioblastoma in establishing orthotopic xenografts, which accurately mirrored paediatric gliomas, making them a suitable model for performing functional studies on paediatric brain tumours [227]. A recent study by Umans R. A. et al. demonstrated the use of zebrafish PDX (zPDX) xenografts to model perivascular glioma invasion through the orthotopic transplantation of tumour cells into transgenic Tg(*fli1*:GFP) casper embryos and validated the pharmacological disruption of glioma cell-vascular interactions by inhibiting the Wnt signalling pathways [228]. Other studies employed the heterotopic zPDX models of rhabdoid tumours, harbouring a *SMARCB1* deletion for the therapeutic evaluation of epigenetically targeted drugs as an efficient treatment option for ceasing tumour growth and progression [229]. To examine the biological consequence of high levels of ΔNp73 expression in GBM tumour growth and pathogenesis, a zebrafish orthotopic xenograft model was used, which faithfully illustrated the significant role of ΔNp73 in driving the malignant growth of this deadly disease [230]. An orthotopic MB zebrafish xenograft model was established to determine the role of astrocytes in MB microenvironment by culturing the MB cells in astrocyte-conditioned media. The xenotransplanted MB cells displayed increased cell protrusion formation related to the elevated CD133 expression [231].

Overall, zebrafish xenograft models of various paediatric tumours, which address diverse questions in brain cancer biology, have been reported here. These studies validate zebrafish xenografts as a unique and robust model in understanding key mechanisms driving paediatric brain tumour pathogenesis as well as serve as a high-potent drug screening platform for evaluating novel therapeutics.

### 2.4. Practical Challenges and Limitations of Genetic, Transgenesis and Transplantation Techniques

The earlier examples of zebrafish paediatric cancer models demonstrate their suitability to recapitulate patient tumour histopathology and reveal their direct clinical implications in developing diagnostic markers and potential therapeutics. Cancer modelling in zebrafish is based on employing multiple genetic tools to replicate patient tumour pathology allowing to understand the molecular drivers and signalling pathways involved in tumour pathogenesis. This is mainly achieved through the cell-type specific expression of human oncogenes using transgenesis techniques, creating the loss-of-function mutations of tumour suppressors facilitated by genome editing or by performing molecular or functional analysis on xenografted human cancer cells. Although a myriad of genetic tools or approaches are available for these techniques, each has its own advantages and limitations. Although ZFNs and TALENs were used initially for paediatric cancer modelling in zebrafish, both these techniques are inefficient, costly, labour-intensive and impose off-targeting effects. Efforts have been made to improve the efficiency and off-targeting effects by generating FokI variants with improved dimerization and cleavage. However, recent advancement in CRISPR/Cas9 system has opened its unprecedented opportunities for precise genome editing in zebrafish, including in the generation of paediatric cancer models. Custom-made CRISPR gRNAs have facilitated the rapid screening of genetic modifiers and tumour suppressors in cancer. Further, the development of CRISPR/Cas9 system for efficient biallelic gene targeting has facilitated the rapid screening of potential identifiers in F0 generation. Furthermore, the CRISPR/Cas9 system has been used for generating tissue-specific knockouts in zebrafish, facilitating the spatial control of gene disruption in somatic cells [83]. However, the CRISPR/Cas9 system also imposes some limitations; the requirement of PAM motif limits the targeting scope, which has been recently addressed by the development of multiple Cas9 variants that have consequently improved target coverage and specificity. Transgenic approaches in zebrafish offer a unique set of tools for understanding specific questions associated with tumour biology. Each of these tools has advantages and limitations based on the experimental goal and technical capabilities. A major offset to the transgenesis technique is the random insertion of the transgene into the genome and leakiness in the spatial and temporal control of the transgene of interest. However, this is addressed by the synergistic application of multiple transgenesis approach to improve the efficiency and to achieve fine tuning on the spatio-temporal control of transgene expression. Xenotransplantation poses temperature-related limitation as the key disadvantage, as the physiological temperature in zebrafish is 28–29 °C compared to 37 °C in humans, requiring zebrafish to be reared at higher temperatures, hampering their normal embryonic development and survival. To address this, xenotransplanted embryos are maintained at 32–34 °C to reflect human body conditions. Overall, each approach provides unique opportunities for understanding the molecular signatures and signalling pathways associated with paediatric brain tumour.

## 3. Comparison between Zebrafish and Other Paediatric Brain Cancer Models

Despite the significant advances and clear benefits that zebrafish tumour models have brought to further our understanding of cancer and its molecular heterogeneity, there still remains a large number of pre-clinical in vivo studies relying on other animal models, such as mouse and rats, as the primary species to study paediatric brain tumours pathophysiology. Similar to zebrafish, the major techniques used in murine models include PDX and transgenic and, in some rare cases, carcinogen-induced models and although these models are used frequently, each of them has its own advantages and limitations [232,233].

PDX is the most widely used method in murine models, which involves transplanting established cancer cells, patient-derived cancer cells or brain tissues extracted directly from the patient into the animals. With all three methods, host animals are primarily immunodeficient (lack functional T and B lymphocytes, NK cells and macrophages), thus ensuring the success of the transplantation and development of the tumour. Using either orthotopic or heterotopic xenograft administration techniques, the growth, progression, invasive, metastatic and response to therapies can be easily monitored in this model by examining changes in tumour volume and spread. Although PDX murine models are popular in paediatric brain cancer studies, it is accompanied by significant limitations, such as the reduced rates of tumour engraftment, which depends greatly on the integrity of the tissue and its handling. Another major limitation includes the time the tissue is obtained (at diagnosis, surgical resection or post-mortem), as it has been shown that molecular features and response to therapies differ depending on the time of tissue collection [234], and a further significant limitation is the use of immunocompromised mice. The lack of an immune microenvironment in these models makes it impossible to study the tumour-immune microenvironment and their interactions [235]. Recently, with the advances in genome editing, the use of genetically engineered animal models has gained a lot of attraction. The ability to study tumour growth and development in a non-immunocompromised system and undisrupted tumour microenvironments has allowed researchers to use these animal models to study a range of different areas in paediatric brain cancers, including tumour pathology and personalised drug discovery, therapy and targeting. However, as with most rodent models, the drawbacks to working with these forms of models include factors such as being costly, technically complicated processes and large variations between individual animals. Table 3 provides some of the models used to study paediatric brain cancers, summarises and compares their advantages and limitations with zebrafish models [232,236].

## 4. Future Directions and Conclusions

Paediatric brain cancers are rare and differ from adult brain tumours in many aspects, including their tumour type, incidence and treatment [237]. Pre-clinical studies that closely mimic paediatric brain tumour physiology are critical in developing effective therapies. Paediatric brain tumour modelling has been challenging to a certain extent due to the sparse availability of paediatric brain tumour cell lines and PDXs. To further add to this complexity, recent advancement in the genomic characterisation and identification of new molecular signatures renders this heterogenic paediatric brain tumour modelling a difficult task. A perfect in vivo paediatric brain tumour model is expected to recapitulate the parental tumour histopathology, molecular features, tumour/host interactions, tumour microenvironment and pharmacokinetic properties in a time and cost-effective manner. In addition to this, the model should have high reproducibility so it can be further used for testing therapeutic drugs. Paediatric brain tumour modelling in rodent models is costly and time-consuming (gestational cycle is 20 days) with PDX implantation in immunodeficient mice taking about 2–8 months for tumour engraftment, before which it is utilized for therapeutic testing. 

Owing to the high physiological and genetic homology to humans, the zebrafish serves as a powerful alternate in vivo model to decipher the molecular mechanisms driving paediatric brain cancers. Unlike mouse models, zebrafish are well-suited for performing the functional characterization of multiple genetic mutations, tumour heterogeneity and for determining the synergy of mutations observed in human tumours. The optical clarity of zebrafish embryos and the transparent adult casper line facilitates in vivo real-time visualisation and monitoring of tumour initiation, tumour cell morphology, motility, metastasis, tumour cell interactions and response to drugs at a single cell resolution. Transgenic zebrafish lines expressing vascular-specific fluorescence facilitate investigations on neo-vascularisation, a pivotal step underpinning the process of tumour growth and proliferation. Progress in the zebrafish cancer modelling approaches from mutagenesis or transgenesis to transplantation techniques has expanded the potential of this in vivo model in paediatric brain tumour studies. Unlike rodent models, paediatric xenograft brain tumour modelling in zebrafish is rapid, with engraftment in embryos observed at 3 dpf following transplantation on 2 dpf embryos, whereas adult Casper *il2rgc.a^−/−^; prkdc^−/−^* strain takes about 7–10 days for tumour engraftment. Zebrafish metastatic heterotopic models and intracranial orthotopic models have added great value in understanding the key processes in the tumour progression as well as in defining the therapeutic inclination of these tumours. The suitability of zebrafish embryos for high throughput drug screening and their unique pharmacokinetic profile further adds to this in vivo model’s arsenal of skills [238]. Studies report the metabolism, distribution and allocation of drugs in zebrafish to be similar to the humans and could thus be scaled to other vertebrates. Zebrafish embryos absorb drugs through water, while adults are orally gavaged. Zebrafish has emerged as a robust model for cancer modelling, with zebrafish PDXs and xenografts making a huge impact in developing anticancer therapies and personalised medicine [206,239]. Delayed adaptive immunity in zebrafish embryos facilitates the xenotransplantation and implantation of various tumours, with their immune cells fully maturing by 3 weeks, allowing a short duration of 7–10 days for monitoring the drug response. 

Despite the plethora of studies on paediatric brain tumour models, thus far there is no single in vivo model that meets all the essential criteria. Zebrafish, similar to other animal models, present some limitations. Genetic differences between humans and zebrafish add a level of complexity to efficient cancer modelling, as teleost-specific genome duplication in zebrafish results in the presence of more than one ortholog for human genes [40]. Gene duplication in zebrafish can often complicate loss-of-function studies on tumour suppressor genes, as these gene orthologs can either have functional overlap allowing them to compensate each other’s loss or hold functionally divergent roles. The majority of the cancer xenotransplantation studies in zebrafish are performed in embryos as studies in adult animals require the use of immunosuppressed animals. In addition, the higher temperature requirement (37 °C) for the transplanted tumour cells hampers the overall embryo survival [198,240]. To overcome these, in 2019, Yan C et al. reported the use of a novel optically clear *prkdc^−/−^ il2rga^−/−^* zebrafish line that lacks T, B and natural killer cells and which has been shown to tolerate temperatures as high as 37 °C [74]. Tumour microenvironment is one other factor that is less explored when using zebrafish cancer models. The use of immunosuppressed or immune-deficient zebrafish reduces the suitability of these models in understanding the tumour microenvironment interactions. However, advancement in the field has resulted in the development of humanised PDX models, where the animals are transplanted with hematopoietic stem cells along with PDX samples. These models are ideal as they allow the assessment of tumour dynamics as well as tumour/immune cell interactions, which is crucial for the development of impactful anticancer immunotherapies [241]. Further, studies using orthotopic xenotransplantation as well as the allotransplantation of paediatric brain tumours into immune-competent zebrafish paves the way for the unravelling of the mechanisms of tumour–microenvironment interactions. Although the pharmacokinetic profiles are similar between zebrafish and humans, there has always been a debate and unacceptance with regard to the scalability of zebrafish drug doses to clinically-relevant dosages. Further, the drugs need to be exposed to embryos via water for an extended period, leaving uncertainties regarding the drug dosage absorbed by the embryos. Inconsistencies with transplantation or microinjection and lack of reproducibility also compromise the widespread use of this model. These limitations can be circumvented by following standard protocols and by using a high level of expertise. Advancements in technology, such as the use of robotics for microinjection and progress in chemical screening, will eventually mark the impeccable role of zebrafish in modelling paediatric brain tumours [242]. In conclusion, zebrafish paediatric brain tumour models offer great value and provide avenues that could solve the unresolved questions in childhood brain cancers.

## Figures and Tables

**Table 2:** Transplantation models of paediatric brain tumours in zebrafish.

Cancer	Injection Site	Cell Line	Species of Origin of Transplanted Cells	Stage	Zebrafish Strain	Ref.
CNS-PNETs	Fourth ventricle	Primary tumours derived from Tg(*sox10*:mCherry-NRASWT)/p53^M214K^	Zebrafish	2 dpf	mitfa^w2^; p53^M214K^	[50,225]
Glioblastoma	Brain ventricle	BPC-A7	Human	2 dpf	WT	[227]
	Intracranial or trunk	D2159MG	Human	3 dpf	Tg(*fli1a*:eGFP)y1;casper or Tg(*glut1b*:mCherry)	[228]
	Midbrain–hindbrain boundary	SJGBM2-Ctr or SJGBM2-ΔNp73	Human	36 hpf	Casper mutants (mitfa−/−; mpv17−/−)	[230]
Medulloblastoma	Hindbrain ventricle	Daoy cells	Human	2 dpf	Tg(flk:mCherry); Absolut+/+ (ednrbl−/− mitfa−/−)	[231]
Pilocytic Astrocytoma	Midline of optic tectum	JHH-NF1-PA1	Human	2 dpf	WT	[226]
Rhabdoid tumour	Yolk sac	INF_R_1288_r1	Human	2 dpf	WT	[229]
Mouse glioma, ependymoma, Choroid plexus Carcinoma	Cerebrum	GBM^ERBB2−RFP^ EP^RTBDN−RFP^, CPC^RFP^	Mouse	30 dpf	WT immunosuppressed	[223]

**Table 3:** Comparison between zebrafish and other paediatric brain cancer models.

Model	Cost	Drug Screening Throughput	Advantages	Disadvantages
Cell cultures	Low	Very High	Rapid growth, robust, easy to maintain, modifiable, immortalized, long-term usage and storage.	Can differ genetically from primary tumours with long-term culturing. Do not have tumour microenvironments.
3D spheroids	Low	Very High	Rapid growth, robust, easy to maintain, modifiable, immortalized, long-term usage and storage. Provides fairly similar physiological characteristics to tumours, such as deregulated metabolism and hypoxic tumour cores.	Can genetically vary with long-term culturing. Provides minimal tumour microenvironments.
Organoids	Medium	High	Provides similar tumour heterogeneity, characteristics and tumour microenvironments to human systems.	Technically difficult to generate, costly and can vary in growth
Drosophila	High	Medium	Can obtain large sample numbers at much lower cost than mouse models, genetic manipulation fast and inexpensive, short generation and life span and have more similar tumour microenvironments to humans than cell culture systems.	Brain pathophysiology, circulatory and respiratory systems substantially different to humans. Drug effects and pharmacodynamics differ to human systems. Immune systems differ.
Rodents	Very High	Low	Most closets system to mimic the tumour microenvironment, genetic alterations and pharmacodynamics as the human system.	Time-consuming, technical expertise is required, lacks immune interactions and PDX are highly variable, depending mainly on tissue integrity.
Zebrafish	Medium	High	Can obtain large sample size, optical transparency in embryos aids with imaging, ease of transplantation, high efficiency in genetic manipulation, rapid tumour engraftment and development of tumours with similar histopathology to humans.	Transplantation studies are limited to embryos and requires immune deficient or immunosuppressed adult animals, and the difference in the ambient temperature between zebrafish and humans and drug dosage in embryos is not clinically relevant.

## Data Availability

Not applicable.
